# Primary Pulmonary Artery Leiomyosarcoma with Pulmonary Metastasis Depicted on Enhanced Computer Tomography: A Case Description and an Analysis of the Literature

**DOI:** 10.3390/jcdd11010001

**Published:** 2023-12-20

**Authors:** Wenzhao Zhang, Peiling Li, Jianqun Yu

**Affiliations:** 1 Department of Radiology, West China Hospital, Sichuan University, 37 Guo Xue Alley, Chengdu 610041, China; 2 Department of Critical Care Medicine, Chengdu Shangjinnanfu Hospital, Chengdu 611730, China

**Keywords:** pulmonary artery leiomyosarcoma, computed tomography, enhanced CT

## Abstract

Pulmonary artery leiomyosarcoma is an extremely rare disease, with only a few dozen cases reported worldwide to date. This disease is easily misdiagnosed as pulmonary thromboembolism, leading to improper treatment and accurate diagnosis in the later stages of the disease. Furthermore, this delayed diagnosis may also be the reason for the extremely high mortality rate of patients suffering from it. Early invasive surgery with the goal of complete surgical resection is the standard treatment method. Chemotherapy and radiation therapy have been tried with variable outcomes.

## 1. Introduction

Primary pulmonary artery sarcoma (PAS) is a rare disease type of which pulmonary artery leiomyosarcoma (PAL) is a very rare pathological type, with an incidence rate of 0.001–0.03% [1,2]. Leiomyosarcoma is a malignant mesenchymal tumor often originating from smooth muscle cells mainly in the uterus and gastrointestinal tract. Leiomyosarcoma originating from the pulmonary artery is extremely rare and usually occurs in middle-aged women [3]. In addition, PAL has non-specific clinical signs, including hemoptysis, cough, chest pain, and chest tightness, so it is often misdiagnosed as a chronic pulmonary embolism in clinical work [4,5]. PAL is often detected incidentally after clinical surgery and postmortem. Although the prognosis of primary PAL is poor, the early detection and timely surgical resection of lesions may significantly improve the survival rate of patients [6].

To date, a total of 18 cases of PAL have been reported in English language articles in the PubMed database [1,2,4,7,8,9,10,11,12,13,14,15,16,17,18,19,20] and research on PAL is limited. The computed tomography (CT) findings and differential diagnosis, treatment, and outcome of PAL have not been thoroughly investigated. Herein, we report a case of PAL depicted on enhanced CT images and its treatment and review previous studies so as to provide a comprehensive understanding of PAL. 

## 2. Case Report

**Figure 1:**
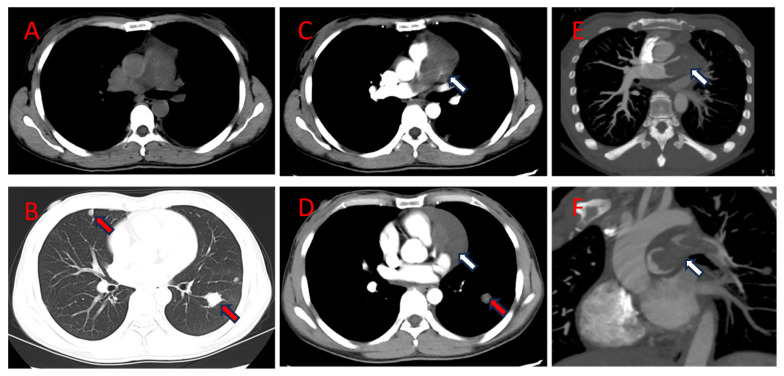
The first chest CT scan of the patient (29 March 2022). Axial CT (**A**–**D**) and MIP (**E**,**F**) showed an irregular soft tissue mass in the pulmonary artery and anterior mediastinum (**white arrow**) that had obvious heterogeneous enhancement (**B**,**D**). Multiple metastatic tumors in both lungs (**red arrow**). CT, computed tomography; MIP, maximum intensity projection.

All procedures performed in this study were in accordance with the ethical standards of the institutional and/or national research committee(s) and with the Helsinki Declaration (as revised in 2013). Written informed consent was provided by the patient for the publication of this case report and the accompanying images. A copy of the written consent is available for review by the editorial office of this journal. A 39-year-old Chinese female patient, who had previously maintained good health, had experienced chest tightness with coughing, chest pain, tightness of breath, and other symptoms of discomfort without inducement for three months and visited the local hospital for examination. A tumor was detected in her anterior mediastinum (no relevant report available). Subsequently, the patient visited the West China Hospital, Sichuan University, for an enhanced CT scan of the chest that revealed a large soft tissue mass in the pulmonary trunk with an approximate size of 4.2 × 2.6 cm. The mass had an irregular shape and heterogeneous enhancement, so it was considered a malignant tumor of the pulmonary artery. The tumor extended to the left and right main pulmonary arteries and resulted in extensive intraluminal filling defects in the pulmonary trunk as well as the right and left main pulmonary arteries and had invaded the mediastinum as well as the adjacent pericardium and cavity of the heart. Multiple metastatic tumors were revealed in both lungs and the pleura (Figure 1) simultaneously. A cardiac color Doppler ultrasound showed that cardiac tumors had invaded the main pulmonary artery and left and right branch veins; right pulmonary artery stenosis and left pulmonary artery occlusion were also observed. Since the onset of the disease, the patient’s blood pressure had increased over one week, with a maximum of 150/80 mmHg and no other symptoms of discomfort.

**Figure 2:**
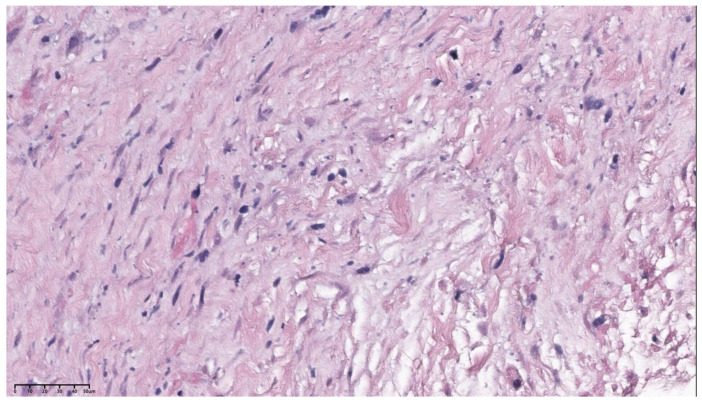
The pathological results (hematoxylin-eosin staining, ×47.3) show that the pulmonary artery tumor represented a leiomyosarcoma histologically.

The pathological diagnosis of the patient’s pulmonary artery mass puncture biopsy was a spindle cell tumor with necrosis; immunohistochemical (IHC) staining and in situ hybridization showed that the tumor cells were SMA (positive), Desmin (positive), Caldesmon (negative), CD34 (negative), S-100 (negative), TLE-1 (partial positive), ER (negative), PR (negative), and WT-1 (negative), with a Ki-67 positive rate of approximately 12–15% and EBER1/2-ISH (negative), which indicated leiomyosarcoma (Figure 2). Combined with imaging and pathological findings, the patient was diagnosed with PAL.

**Figure 3:**
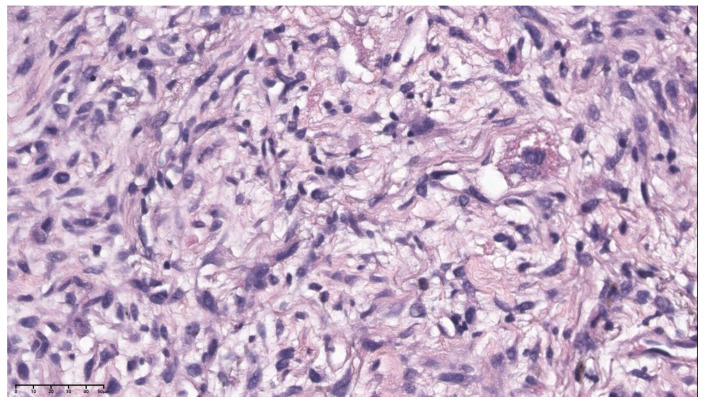
The pathological results (hematoxylin-eosin staining, ×55.8) indicate that the left upper lobe nodule was a metastasis of a pulmonary artery tumor.

The needle biopsy of the left upper lobe nodules combined with IHC staining showed that the spindle cells were PCK (negative), ck7 (negative), P63 (negative), TTF-1 (negative), SMA (partial positive), CD34 (negative), CK8/18 (negative), STAT-6 (negative), ALK-1 (negative), S-100 (negative), Desmin (partial positive), and had Ki-67 positive rates of approximately 10–12%. Based on these findings, a further diagnosis of metastasis or the involvement of pulmonary artery mass lesions was made (Figure 3).

**Figure 4:**
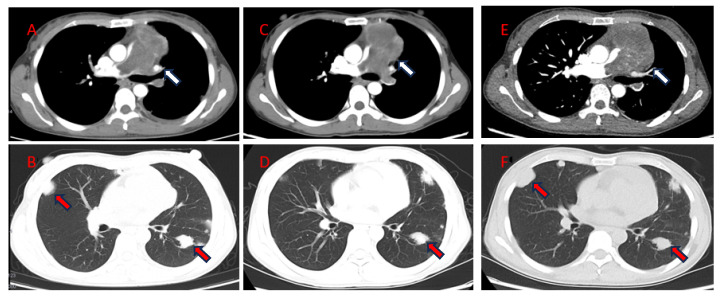
The second CT examinations (**A**,**B**) (12 May 2022), the third CT examinations (**C**,**D**) (3 July 2022), and the fourth CT examinations (**E**,**F**) (26 July 2022) showed that the volume of soft tissue masses in the anterior mediastinum and pulmonary artery had not changed (**white arrow**) and the volume of metastatic tumors in both lungs had decreased during chemotherapy (**red arrow**). CT, computed tomography.

Combined with the results of imaging and the pathological examination, a diagnosis of PAL with bilateral lung metastasis was made. A chest CT performed two months later showed that the pulmonary artery lesions were significantly enlarged and metastatic tumors had enlarged and increased in number in both lungs compared with before. The patient subsequently accepted three cycles of GT regimen chemotherapy (gemcitabine + docetaxel). After she had finished the first and third cycles of treatment, she underwent an additional chest CT scan, respectively, which showed that the patient’s pulmonary artery lesions had not changed but that the lung metastases had shrunk and decreased (Figure 4).

**Figure 5:**
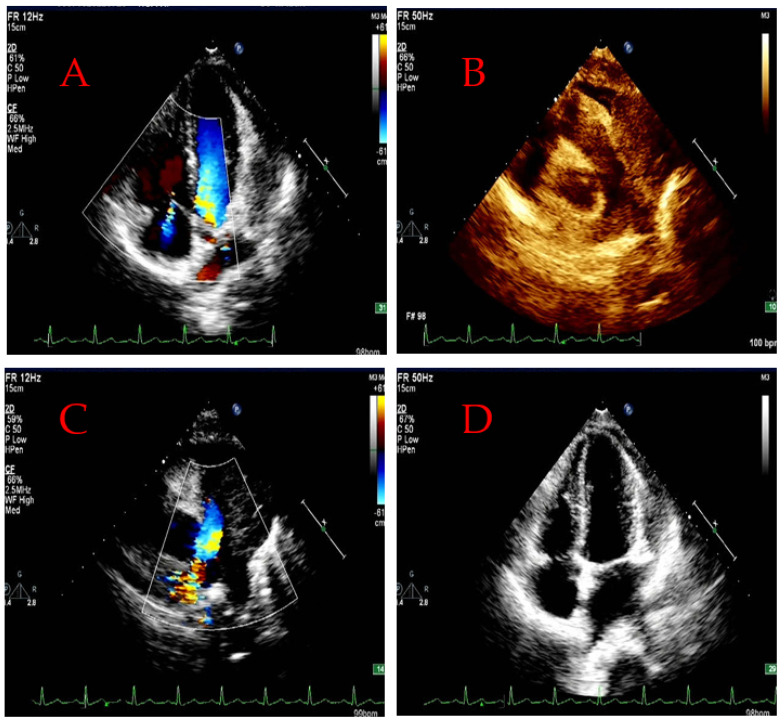
The patient’s echocardiography (**A**–**D**) (28 July 2022) showed that in the mediastinal and pulmonary artery masses tricuspid valve regurgitation (very mild), pulmonary hypertension (moderate), and biventricular systolic function were normal.

**Figure 6:**
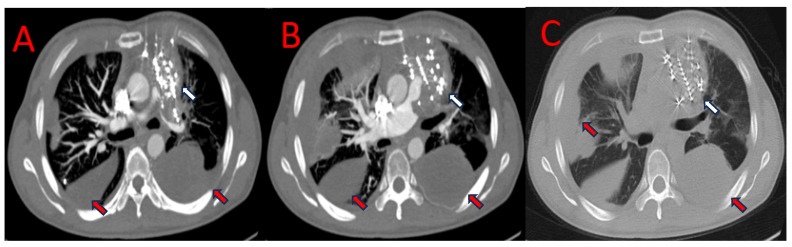
The fifth chest CT scan of the patient in West China Hospital, Sichuan University (6 February 2023). MIP (**A**,**B**) and axial CT (**C**) showed that after radioactive particle implants had been performed (**white arrow**) the volume of soft tissue masses in the pulmonary artery and anterior mediastinum decreased, but bilateral lung and pleural metastases increased significantly (**red arrow**). CT, computed tomography; MIP, maximum intensity projection.

The patient underwent echocardiographic examination at the same time. A large solid mass with a size of approximately 69 × 62 mm was found in the inner and outer upper parts of the pulmonary artery, invading the main pulmonary artery and the left and right pulmonary arteries. The boundary of the left wall of the pulmonary artery was unclear. No blood flow signal was observed in the left pulmonary artery, yet a fine bundle blood flow signal was observed in the right pulmonary artery. The blood flow accelerated significantly at Vmax (**Maximum velocity**) = 3.3 m/s and pressure gradient PG = 44 mmHg. The size of each atrium in the heart was normal. The diameter of the aorta was normal. The thickness and amplitude of the interventricular septum and the left ventricular posterior wall were normal. Doppler detection revealed the following: minimal mitral regurgitation and very little tricuspid valve regurgitation at Vmax (**Maximum velocity**) = 3.5 m/s and pressure gradient (PG) = 50 mmHg. No definite shunt was observed in the heart. Tissue Doppler examination revealed a mitral annulus diastole motion spectrum of e ‘>a’. The measurement of biventricular systolic function was normal (Figure 5). At six months after the end of chemotherapy, the lesions of PAL were subjected to radioactive particle implants. The follow-up chest CT examination showed that both the lesion in the pulmonary artery and the filling defect in the lumen of the pulmonary artery were reduced, but the metastatic tumors in both lungs and bilateral pleura were significantly increased and enlarged (Figure 6). Radioactive particle implants therapy, as one of the important local alternative treatments for advanced non-small cell lung cancer, has the characteristics of small trauma, low dosage of radioactive elements, and sustainable irradiation, which can compensate for the shortcomings of surgical treatment and chemotherapy [21]. At present, clinical research on radioactive particle implants therapy mainly focuses on non-small cell lung cancer and there have been few reports on its effects on rare lung cancer types such as small cell lung cancer [22]. The treatment of primary pulmonary artery sarcoma such as PAL has never been reported. In this case report, due to the patient missing the optimal timing for surgical treatment at their initial visit, the use of radioactive particle implants therapy was also a bold attempt; however, the patient’s current treatment effect is, unfortunately, not satisfactory. At the time of writing, the patient is currently undergoing treatment.

## 3. Discussion and Conclusions

We summarized the gender, age, symptoms, and initial diagnosis results of PAL in 18 cases from previous 17 English language articles in the PubMed database [1,2,4,7,8,9,10,11,12,13,14,15,16,17,18,19,20]. The results are shown in Table 1. The incidence rate of males is about 33% (6/18) and the average age is about 53.8 years, while the incidence rate of female is about 67% (12/18) and the average age is about 55.8 years old. The lesion of PAL most frequently occurs in the right pulmonary arteries; for a total of nine patients, the incidence rate is about 50% (9/18). Regarding the main pulmonary arteries, for a total of eight patients the incidence rate is about 44.4% (8/18). Furthermore, regarding left pulmonary arteries, for a total of seven patients the incidence rate is about 38.9% (7/18). However, it is also reported to occur in the right ventricular outflow tract for a total of one patient in which case the incidence rate is about 5.6% (1/18) (Table 1). In both this case and the data above, PAL commonly appears in middle-aged women.

The clinical symptoms for patients with PAL are usually nonspecific, mainly including dyspnea and chest pain, making the early diagnosis of PAL difficult. Before a more accurate diagnosis is achieved through biopsy or surgery, PAL is frequently misdiagnosed as pulmonary embolus (72.2%, 13/18), a tumor (22.2%, 4/18), and mononucleosis (5.6%, 1/18) (Table 1). Mediastinal mass, pulmonary stenosis, and lung cancer are other diagnostic errors [6]. Pulmonary embolism is the most common misdiagnosis; patients may undergo a period of conventional treatment such as anticoagulation and, when no effects are observed with such an intervention, other diseases begin to be considered, thereby missing the opportunity for early treatment [1]. PAL does not have specific imaging signs, but enhanced CT examinations play an important role in determining the nature of the lesion and dynamic observation [23]. On enhanced CT images, chronic pulmonary thromboembolism usually shows abrupt vascular narrowing and cut-offs instead of a continuous soft tissue filling of the pulmonary arteries as the neoplasm [24]. The enhancement of a tumor versus the non-enhancement of an embolus as well as the distension of a vascular lumen by a tumor and the extravascular invasion into the adjacent structure are also important clues to distinguish between tumors and a pulmonary embolism [23]. Increased uptake in the area of the tumor on fluorine-18-2-fluoro2-deoxy-d-glucose positron emission tomography (FDG-PET) can also be helpful for differentiating a neoplasm from an organizing embolus [25].

It has been revealed that PAL may originate from the smooth muscle of the pulmonary parenchyma, pulmonary arteries, and bronchi [26]. PAL can be divided into the intraluminal type, intrapulmonary type, or pulmonary vascular type, according to the location of the lesion, of which the intrapulmonary type is the most common [2]. PAL often grows along the vascular wall, which can lead to pulmonary artery stenosis or obstruction and is easily misdiagnosed as a pulmonary embolism [7]. PAL does not easily metastasize in the early stage, which highlights the importance of early detection [27]. The surgical resection is still the main treatment for PAL [28]. However, other researchers have asserted that patients with PAL treated with surgical resection often experience tumor recurrence [8].

The main feature of PAL is its hard white/gray surface. Under microscopic observation, most tumors are composed of interwoven spindle shaped cell bundles, which have elliptical vesicular nuclei and varying degrees of nuclear atypia often accompanied with signs of bleeding or necrosis. Preoperative bronchoscopy, sputum smear, and lung biopsy are usually negative. Diagnosis can be confirmed through an intraoperative frozen section biopsy or postoperative pathological and immunohistochemical examination. When the immunohistochemical results of actin, SMA, and desmin are positive, the tumor is considered to originate from a smooth muscle. When CD99 is negative, Ewing’s sarcoma should not be considered. Negative EMA results indicate that tumors usually do not originate from epithelial tissue. When S100 is negative, it is not recommended to diagnose a nerve tissue tumor.

Metastasis of PAL is uncommon and typically occurs late in the disease process, which highlights the importance of early detection [2]. According to the previous literature reports, it is recommended that patients with incomplete resection and malignant tumors should be treated with radiotherapy and chemotherapy and the survival period of patients can be extended to 20 months through receiving adjuvant chemotherapy and radiotherapy after surgery [29]. If the patient can undergo complete resection at the early stage of the tumor, the five-year survival rate is close to 50% and there are reports of 20-year survival rates after resection [1,2,4,7,8,9,10,11,12,13,14,15,16,17,18,19,20].

From 1990 to 2023, only one patient has been diagnosed with PAL pathologically in our hospital. Although this PAL patient had mild clinical symptoms, the disease had a short onset and progressed rapidly. At the initial visit to our hospital, the lesion of this patient was already very large, had invaded the main trunk and left branch of the pulmonary artery, and had penetrated outward and involved the anterior and middle mediastinum areas, resulting in the inability to completely remove the tumor. At the same time, due to the multiple metastases of both lungs, the patient did not have indications for surgical treatment. Although the patient accepted the adjuvant chemotherapy, she had a poor prognosis.

In summary, PAL is a rare primary malignant tumor of the pulmonary artery and has no specific clinical symptoms. PAL has enhanced CT features of the malignant tumor, a high degree of malignancy, and rapid disease progression. CT features suggesting malignancy include extensive intraluminal filling defect, mediastinal or pericardial invasion, and metastasis. By combining histological and immunohistochemical markers, the tumor can be correctly diagnosed.

## Figures and Tables

**Table 1:** PAL patient characteristics according to the previous literature reports.

Variables	Number of Cases		Proportion
**Sex**		average age (years)	
Male	6	53.8	33%
Female	12	55.8	67%
**S** **ymptom**			
Dyspnea	11	the incidence rate of female is about 67%	61.1%
Tussis	3		16.7%
Low grade fever	1		5.6%
Thoracalgia	5		27.8%
Apopsychia	1		5.6%
Dizziness	1		5.6%
Weakness	2		11.1%
Acute right heart failure	1		5.6%
Asymptomatic	1		5.6%
**Preliminary diagnosis**			
Pulmonary embolus	13		72.2%
Mononucleosis	1		5.6%
Tumors	4		22.2%
**Location of lesion involved**			
The main pulmonary arteries	8		44.4%
The right pulmonary arteries	9		50.0%
The left pulmonary arteries	7		38.9%
Right ventricular outflow tract	1		5.6%

## Data Availability

We do not share our data.
